# 9,10-Dihydrophenanthrene with Two Spiro(dibenzocycloheptatriene) Units: A Highly Strained Caged Hydrocarbon Exhibiting Reversible Electrochromic Behavior

**DOI:** 10.3390/molecules22111900

**Published:** 2017-11-04

**Authors:** Yusuke Ishigaki, Yuki Hayashi, Kazuma Sugawara, Takuya Shimajiri, Wataru Nojo, Ryo Katoono, Takanori Suzuki

**Affiliations:** Department of Chemistry, Faculty of Science, Hokkaido University, Sapporo 060-0810, Japan; gaudryceras@eis.hokudai.ac.jp (Y.H.); kazuama_ns0210@eis.hokudai.ac.jp (K.S.); t.shimajiri@sci.hokudai.ac.jp (T.S.); nojo@sci.hokudai.ac.jp (W.N.); katoono@sci.hokudai.ac.jp (R.K.)

**Keywords:** polycyclic aromatic hydrocarbons, long bond, dynamic redox system, dication, dibenzotropylium, electrochromism, through-bond interaction

## Abstract

The title dispiro hydrocarbon **1** was designed as a new electrochromic material. This multiply clamped hexaphenylethane-type electron donor was prepared from 2,2′-diiodobiphenyl via biphenyl-2,2′-diylbis(dibenzotropylium) **2**^2+^ salt. X-ray analysis of **1** revealed a highly strained structure as reflected by an elongated “ethane” bond [bond length: 1.6665(17) Å] and nearly eclipsed conformation. The weakened bond was cleaved upon two-electron oxidation to regenerate the deeply colored dication **2**^2+^. The reversible interconversion between **1** and **2**^2+^ is accompanied not only by a drastic color change but also by C–C bond formation/cleavage. Thus, the voltammogram showed a pair of well-separated redox waves, which is characteristic of “dynamic redox (*dyrex*)” behavior. The tetrahydro derivative of **1** with two units of spiro(dibenzocycloheptadiene), which suffers from more severe steric congestion, was also prepared. The crystallographically determined bond length for the central C–C bond [1.705(4) Å] is greatest among the values reported for 9,9,10,10-tetraaryl-9,10-dihydrophenanthrene derivatives.

## 1. Introduction

*π*–Conjugated systems have attracted much attention due to the facile modification of their properties by unique molecular design, as shown in several reports on sterically-congested and/or curved polycyclic aromatic hydrocarbons (PAHs) [1,2,3,4]. Some of them have been shown to have unusual geometric features, such as extremely twisted aromatic rings [5,6,7,8,9,10,11,12] or an extraordinarily long C–C bond [13,14,15,16,17,18,19,20], which result in intriguing physical properties.

9,10-Dihydrophenanthrene (DHP) with four aryl groups at the 9,9,10,10-positions is the clamped derivative of hexaphenylethane (HPE). In contrast to the easy dissociation of unclamped HPEs into two trityl radicals upon cleavage of the “ethane” bond [21], a series of tetraaryl-DHPs (Ar_4_DHPs) are considered to be thermally stable [22]. The C_9_–C_10_ bond fission in Ar_4_DHP is no longer entropically favored, so that even an elongated bond with a bond length far greater than the standard (1.54 Å) is intact when incorporated in the Ar_4_DHP skeleton [23]. Thus, the DHP skeleton is one of the most useful scaffolds for examining highly strained structures and their special properties.

**Figure 1:**
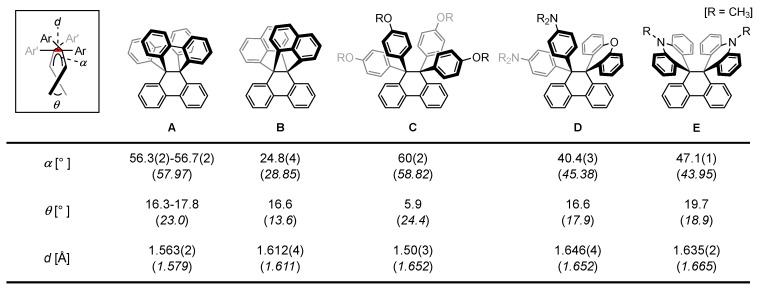
Reported structural parameters of Ar_4_DHPs determined by X-ray analyses. The values obtained by DFT calculations at the B3LYP/6-31G* level in this study are shown in brackets.

Hexabenzo[4.4.4]propellane **A** [24], which was first reported in 1971, is a multiply clamped HPE in which the central Csp^3^–Csp^3^ bond [bond length: 1.563(2) Å] is not greatly expanded [25]. In this nonspiro-type clamped HPE, the steric repulsion among the six benzene rings is effectively reduced by adopting a twisted conformation with a torsion angle (*α*) of about 60° around the central C–C bond (Figure 1). On the other hand, dibenzodinaphtho[4.3.3]propellane **B** [26] has a longer bond [1.612(4) Å] because it has a less-skewed geometry due to the rigid naphthalene planes. This difference in bond length as well as its correlation with torsion/twisting angles (*α*, *θ*) could be supported by DFT calculations. On the other hand, Ar_4_DHPs were predicted to have much longer C–C bonds than those in **B** since the steric hindrance regarding the C_9_–C_10_ bond is greater for two unfused benzene rings than for a naphthalene nucleus.

We previously studied the structures and properties of a series of Ar_4_DHPs [27,28,29,30,31,32]. When we conducted an X-ray analysis of (4-CH_3_OC_6_H_4_)_4_DHP (**C**), we could not obtain accurate values for the bond length or torsion/twisting angles due to the positional disorder of the ethane unit [27]. Such disorder has often been observed in globular ethanes (e.g., unclamped HPEs [33] or hexachloroethane [34]), but was not present in the spiro-type Ar_4_DHPs (**D** and **E**). Their C_9_–C_10_ bond lengths could be accurately determined to be 1.646(4) and 1.635(2) Å by X-ray analyses [29,30]. These values are greater than those for other DHPs, and, thus, the spiro ring can be considered to be the key structure for observing a very long bond in Ar_4_DHPs.

Another interesting point regarding Ar_4_DHP is the electrochromic response with “dynamic redox (*dyrex*)” behavior. Two-electron oxidation induces the formation of cationic chromophores accompanied by fission of the elongated C_9_–C_10_ bond. Heterocyclic units (acridan/xanthene) or alkoxy/amino groups have often been incorporated into Ar_4_DHP to raise the HOMO level and to stabilize the corresponding dicationic species. However, under an appropriate molecular design, we envisaged that reversible electrochromic systems could be constructed without the aid of heteroatoms. Herein we report the details of the title spiro-type Ar_4_DHP **1**, which is a pure hydrocarbon that can still exhibit reversible electrochromic behavior thanks to the raised HOMO level of **1** as well as the stability of the 14*π*–dibenzotropylium unit in the bond-dissociated dication **2**^2+^.

## 2. Results and Discussion

### 2.1. Design and Theoretical Study

**Figure 2:**
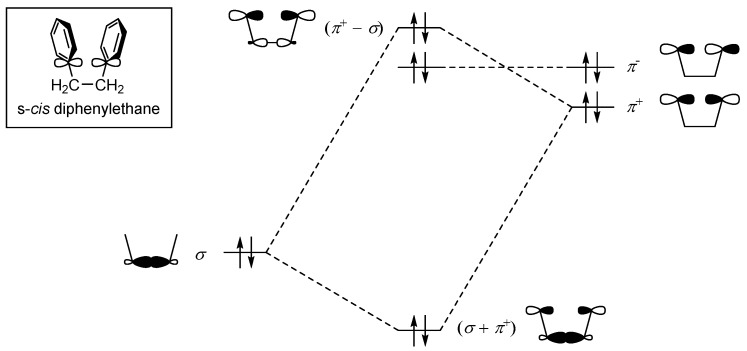
Schematic view of through-bond interaction in the diphenylethane model.

The through-bond interaction (TBI) [35] in PAHs is an important factor which modifies the MO levels that determine their electron-donating properties. As postulated based on a simplified s-*cis* diphenylethane model (Figure 2), the *σ*–orbital of the ethane bond would be lowered whereas the *π*^+^–orbital should be raised through TBI. Greater perturbation is expected when the two energy levels are closer. The parallel arrangement of these orbitals is also important for realizing effective TBI.

**Figure 3:**
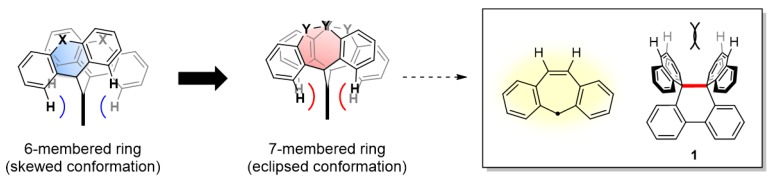
Molecular design of Ar_4_DHP **1** with two spiro(dibenzocycloheptatriene) units.

For Ar_4_DHP, TBI would be maximized by elongation of the C_9_–C_10_ bond, since a longer bond has a higher *σ*–orbital level to narrow the energy gap toward the *π*^+^–orbital. At the same time, an eclipsed conformation is desirable to ensure that the orbitals are parallel. The spiro(dibenzocycloheptatriene) units are the ideal skeleton to be incorporated into Ar_4_DHP. In general, Ar_4_DHPs tend to adopt a skewed geometry to reduce the “front strain” [21] among the four aryl groups over the C_9_–C_10_ bond. The spiro(xanthene or 10-methylacridane) with the central six-membered ring can force the DHP skeleton to adopt a less-skewed geometry (Figure 1). Here, we propose that spiro(dibenzocycloheptatriene) with a central seven-membered ring is more favorable since it can make the DHP skeleton become nearly eclipsed by the greater steric hindrance between the inner protons on the benzo groups and the DHP plane (Figure 3). The nearly eclipsed conformation would also cause elongation of the C_9_–C_10_ bond due to the greater steric repulsion than in the skewed conformation, and, thus, the HOMO level of Ar_4_DHP with two spiro(dibenzocycloheptatriene) **1** would be effectively raised through TBI.

The tetrahydro derivative **3** with two units of spiro(dibenzocycloheptadiene) was also included in this study as a reference compound; it cannot adopt a similar eclipsed conformation due to severe steric repulsion between the CH_2_CH_2_ units in the cycloheptadiene rings.

**Figure 4:**
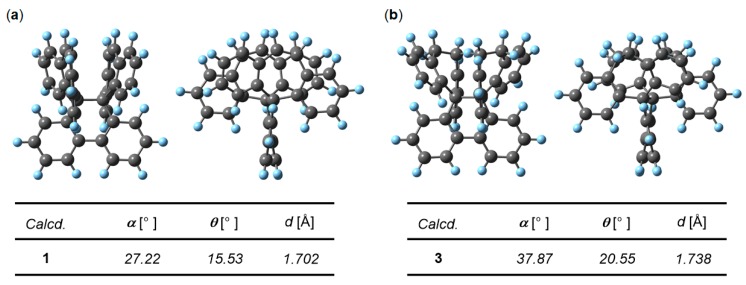
Optimized structures (left: front view, right: side view) of (**a**) **1** and (**b**) **3**.

Density functional theory (DFT) calculations [36] at the B3LYP/6-31G* level predicted that the optimized structure of **1** adopts a nearly eclipsed conformation, as designed. The C_9_–C_10_ bond length was estimated to be greater than 1.7 Å, which is much greater than the values previously reported for other Ar_4_DHPs (Figure 4). The calculated HOMO level of **1** was raised to −5.25 eV, which is much higher than that of (4-CH_3_OC_6_H_4_)_4_DHP (−5.44 eV). Thus, we designed a stronger electron-donating hydrocarbon than Ar_4_DHP with four CH_3_O groups. 

**Figure 5:**
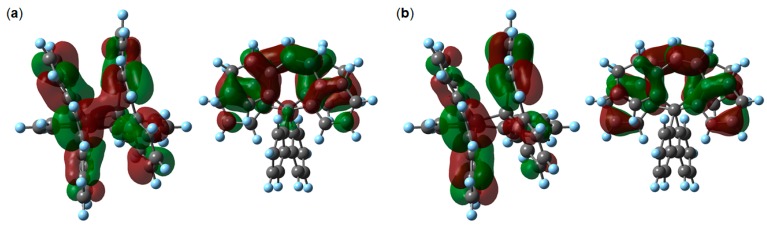
(**a**) HOMO and (**b**) next-highest OMO (NHOMO) of **1** based on DFT calculations (B3LYP/6-31G*).

As shown in Figure 5, the HOMO of **1** has the character of (*π*^+^ – *σ*), as evidenced by the large orbital coefficients on the ethane bond. Thus, the effective TBI between *π*^+^ and *σ* causes the perturbation of both orbital levels, resulting in an increase in the energy of (*π*^+^ – *σ*) to become higher than that of *π*^−^ [next-highest OMO (NHOMO), −5.39 eV].

### 2.2. Preparation and X-ray Analysis

**Scheme 1:**
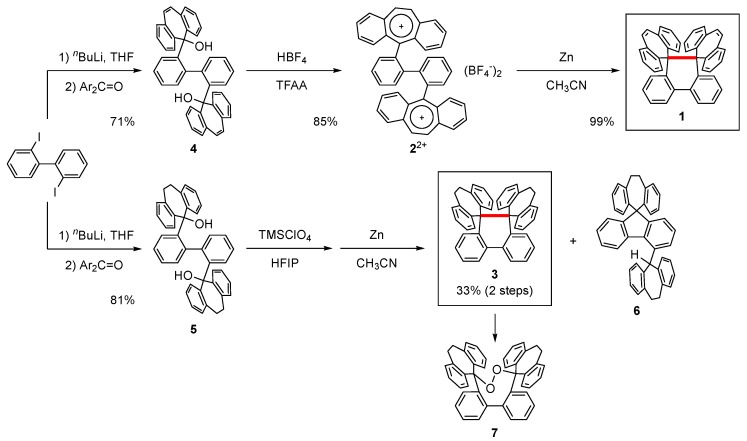
Preparation of newly designed Ar_4_HPEs **1** and **3**.

With 2,2′-diiodobiphenyl as a starting material, diol **4** was prepared by successive reactions with BuLi and dibenzotropone [37]. Upon treatment of **4** with HBF_4_ in trifluoroacetic anhydride (TFAA), the dication salt **2**^2+^(BF_4_^−^)_2_ was isolated as a deep purple powder with 85% yield. When dication **2**^2+^ was reduced with Zn powder, the newly designed dispiro Ar_4_DHP **1** with two spiro(dibenzocycloheptatriene) units was obtained quantitatively as a white solid (Scheme 1).

In the case of the spiro(dibenzocycloheptadiene) derivative, diol **5** was obtained in a similar manner and treated with TMSClO_4_ [38] in 1,1,1,3,3,3-hexafluoro-2-propanol (HFIP). The resulting precursor dication was directly reduced with Zn powder to give the desired compound **3** in 33% yield as a white solid. The low yield of **3** can be partly explained by the formation of by-products/decomposition products. From the mixture, isomer **6** with a spiro(fluorene) framework was isolated. The eight-membered-ring endoperoxide **7** was also generated as a decomposition pathway of **3**. 

**Figure 6:**
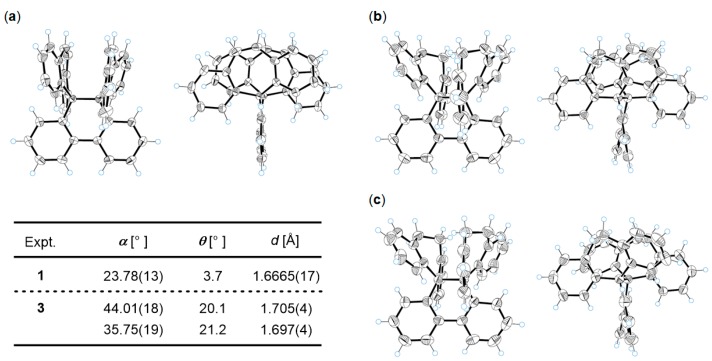
ORTEP drawings (left: front view, right: side view) of (**a**) **1**; (**b**) **3** (mol-1) and (**c**) **3** (mol-2) at 150 K. Thermal ellipsoids are shown at the 50% probability level.

X-ray analyses were performed at 150 K by using single crystals of **1** and **3** (Figure 6). Consistent with the results of DFT calculations, **1** adopts a nearly eclipsed geometry. For the DHP skeleton, the torsion angle *α* over the C_9_–C_10_ bond is 23.78(13)° and the dihedral angle *θ* for the biphenyl unit is only 3.7°. Due to the lack of skewing deformation to reduce the “front” strain, the C_9_–C_10_ bond is expanded. The length of 1.6665(17) Å is greater than any value ever reported for Ar_4_DHPs [39].

As indicated by the DFT calculation shown in Figure 4, both of the crystallographically independent molecules of **3** adopt a twisted conformation. In this way, close contact between the two CH_2_CH_2_ units over the C_9_–C_10_ bond is avoided. However, the molecules still suffer from large steric hindrance between the two spiro(dibenzocycloheptadiene) units. Thus, the bond lengths for the C_9_–C_10_ bond [1.705(4) and 1.697(4) Å] of **3** are the highest values reported for the Ar_4_DHP, including **1**.

### 2.3. Redox Behavior 

**Figure 7:**
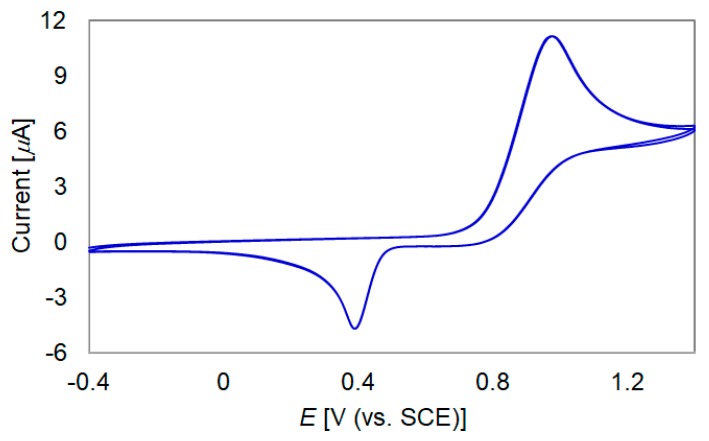
Cyclic voltammogram of **1** in CH_2_Cl_2_ containing 0.1 M Bu_4_NBF_4_ as a supporting electrolyte (scan rate 100 mV s^−1^, Pt electrode).

To investigate the electron-donating properties and reversibility of the redox behavior of **1**, redox potentials were measured by cyclic voltammetry in CH_2_Cl_2_ (Figure 7). The one-wave two-electron oxidation peak was observed, which corresponds to the formation of the dication **2**^2+^ accompanied by C_9_–C_10_ bond cleavage. The corresponding reduction peak of **2**^2+^ appeared at +0.42 V. Such a separation of redox peaks is characteristic of *dyrex* systems. When scanning was repeated twice, no change was observed in the voltammogram, which indicates highly reversible interconversion between **1** and **2**^2+^.

The electron-donating properties of hydrocarbon **1** are striking. The oxidation potential (+0.95 V) is far less positive than that of (4-CH_3_OC_6_H_4_)_4_DHP (+1.44 V) measured under similar conditions [28]. Such a change can be qualitatively accounted for by the different HOMO levels calculated by the DFT method, which demonstrates the validity of our concept for raising the HOMO level of Ar_4_DHP through TBI without the aid of heteroatoms.

### 2.4. Electrochromic Behavior

**Figure 8:**
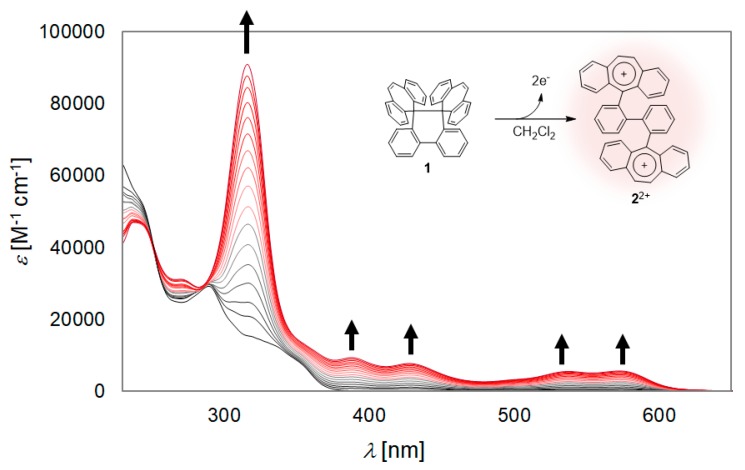
Change in the UV/Vis spectrum upon electrochemical oxidation (20 µA) of **1** in CH_2_Cl_2_ containing 0.05 M Bu_4_NBF_4_ as a supporting electrolyte (every 4 min).

Upon the electrochemical oxidation of **1** in CH_2_Cl_2_, from colorless to red, an electrochromic response was observed with an isosbestic point at 252 nm. The final UV/Vis spectrum is identical to that of the isolated dication **2**^2+^(BF_4_^−^)_2_ (Figure 8). Regeneration of **1** with a drastic color change from red to colorless was attained by reverse electrolysis of the as-prepared dicationic solution (see Supplementary Materials Figure S1). This is a rare successful demonstration of electrochromic behavior based on pure hydrocarbon redox species [40].

**Figure 9:**
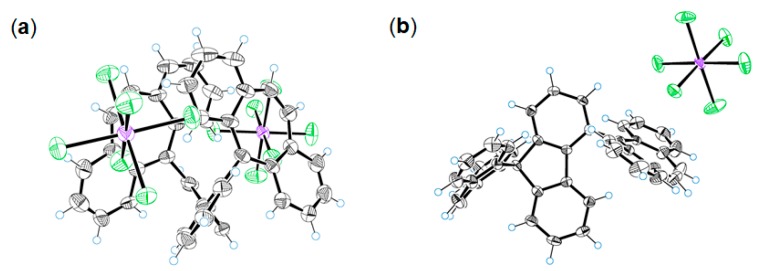
ORTEP drawings of (**a**) **2**^2+^(SbCl_6_^−^)_2_·2(CH_2_Cl_2_) and (**b**) **8**^+^(SbCl_6_^−^)·(CH_3_CN) at 150 K. Solvent molecules are omitted for clarity and thermal ellipsoids are shown at the 50% probability level.

Quantitative interconversion between donor **1** and dication **2**^2+^ can be conducted in a preparative manner by chemical oxidation and reduction. Thus, upon treatment of **1** with two equivalents of (4-BrC_6_H_4_)_3_N^+●^SbCl_6_^−^ in CH_2_Cl_2_, dication **2**^2+^ was isolated quantitatively as a stable salt. The X-ray structural analysis of **2**^2+^(SbCl_6_^−^)_2_·2(CH_2_Cl_2_) crystal shows that there is a *π*–*π* stacking interaction between two dibenzotropylium units [dihedral angle: 12.1°; closest C–C contact: 3.31(2) Å] (Figure 9a). The biphenyl unit in **2**^2+^ is twisted by 70.6°.

**Figure 10:**
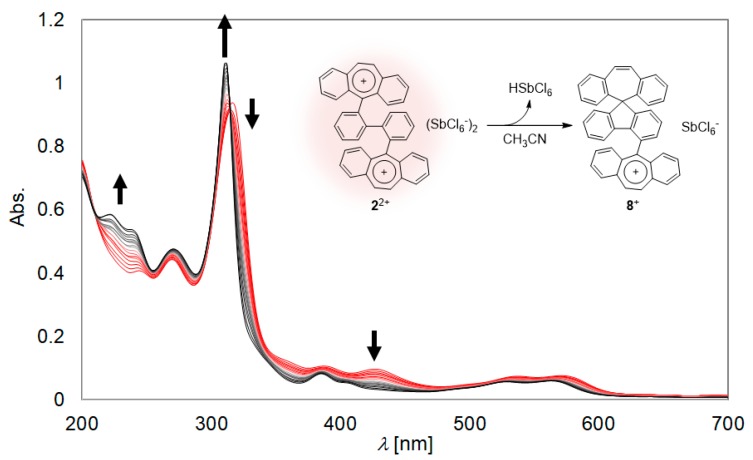
A change in the UV/Vis spectrum of **2**^2+^ upon standing at 23 °C in CH_3_CN (0–12 min, every 2 min; 12–36 min, every 4 min; 36–52 min, every 8 min; 52–84 min, every 16 min).

While **2**^2+^ was persistent in CH_2_Cl_2_, it underwent faci transformation into spiro(fluorene)-type monocation **8**^+^ in CH_3_CN. When this conversion was followed by UV/Vis spectroscopy at 23 °C, several isosbestic points were observed (Figure 10), from which a reaction rate of 5.0 × 10^−4^ s^−1^ was deduced. Friedel–Crafts-type cyclization also proceeded cleanly in a preparative scale, and **8**^+^ was isolated as a stable salt with 94% yield. The spiro-structure was unambiguously determined by X-ray analysis of the **8**^+^(SbCl_6_^−^)·(CH_3_CN) crystal (Figure 9b).

Many dicationic dyes with a biphenyl-2,2′-diyl skeleton adopt a twisted structure similar to that of **2**^2+^ with a *π*–*π* stacking arrangement, for which solvent polarity affects the twisting angle: more acute in a non-polar/less polar solvent and nearly perpendicular in a polar solvent [41,42]. The observed solvent effects for the Friedel–Crafts-type cyclization of **2**^2+^ might be related to this difference in dication conformation depending on the solvent. Facile degradation to the monocation **8**^+^ in CH_3_CN may be related to the largely twisted conformation to facilitate nucleophilic addition of a benzene ring to the cationic center. The lack of further spiro cyclization of **8**^+^ might be related to the ring strain of the 4,8-dihydrocyclopenta[*def*]fluorene structure [43].

## 3. Experimental

### 3.1. General Methods

All reactions were carried out under an argon atmosphere. All commercially available compounds were used without further purification unless otherwise indicated. Dry CH_3_CN was obtained by distillation from CaH_2_ prior to use. Column chromatography was performed on silica gel I-6-40 (YMC) of particle size 40–63 µm. ^1^H and ^13^C NMR spectra were recorded on a Ascend^TM^ 400 (^1^H/400 MHz and ^13^C/100 MHz) spectrometer (BRUKER, Billerica, MA, USA). IR spectra were measured as a KBr pellet on a JIR-WINSPEC100 FT/IR spectrophotometer (JEOL, Tokyo, Japan). Mass spectra were recorded on a JMS-T100GCV spectrometer (JEOL, Tokyo, Japan) in FD mode (GC-MS&NMR Laboratory, Research Faculty of Agriculture, Hokkaido University). Melting points were measured on a Yamato MP-21 or Yanagimoto micro melting point apparatus and are uncorrected. UV/Vis spectra were recorded on a U-3500 spectrophotometer (Hitachi, Tokyo, Japan). DFT calculations were performed with the Gaussian 09W program package (Revision E.01, 2013, Gaussian Inc., Wallingford, CT USA). The geometries of the compounds were optimized by using the B3LYP method in combination with the 6-31G* basis set. Redox potentials (*E*^ox^ and *E*^red^) were measured by cyclic voltammetry in dry CH_2_Cl_2_ containing 0.1 M^−^^1^ Bu_4_NBF_4_ as a supporting electrolyte. All of the values shown in the text are in *E*/V versus SCE measured at a scan rate of 100 mV s^−^^1^. Pt disk electrodes were used as the working and counter electrodes. The working electrode was polished using a water suspension of aluminum oxide (0.05 µm) before use. The irreversible half-wave potentials were estimated from the anodic peak potentials (*E*_pa_) as *E*^ox^ = *E*_pa_ − 0.03 or the cathodic peak potentials (*E*_pc_) as *E*^red^ = *E*_pc_ + 0.03.

### 3.2. Synthetic Procedures

#### 3.2.1. Dispiro(dibenzo[*a*,*d*]cycloheptatriene-5,9′-phenanthrene-10′,5″-dibenzo[*a*,*d*]cycloheptatriene) **1**

From **2**^2+^(SbCl_6_^−^)_2_: To a solution of **2**^2+^(SbCl_6_^−^)_2_ (46.3 mg, 38.5 µmol) in dry CH_3_CN (1.8 mL) was added activated zinc powder (50.7 mg, 775 µmol) at 25 °C. The mixture was stirred for 15 min, and then diluted with water. The whole mixture was extracted with CH_2_Cl_2_ three times. The combined organic layers were washed with water and brine, and dried over anhydrous Na_2_SO_4_. After filtration, the solvent was concentrated under reduced pressure. The crude product was purified by column chromatography on silica gel (CH_2_Cl_2_) to give **1** (20.4 mg) as a white solid with 100% yield.

From **2**^2+^(BF_4_^−^)_2_: To a solution of **2**^2+^(BF_4_^−^)_2_ (265 mg, 375 µmol) in dry CH_3_CN (8.5 mL) was added activated zinc powder (496 mg, 7.59 mmol) at 24 °C. The mixture was stirred for 1 h at 24 °C, and then diluted with water. The whole mixture was extracted with CH_2_Cl_2_ three times. The combined organic layers were washed with water and brine, and dried over anhydrous Na_2_SO_4_. After filtration through silica gel, the solvent was concentrated under reduced pressure to give **1** (198 mg) as a white solid with 99% yield.

Mp: 259–260 °C (decomp.); ^1^H NMR (CDCl_3_): *δ*/ppm 8.10 (2H, dd, *J* = 1.2, 8.2Hz), 7.33 (2H, ddd, *J* = 1.3, 7.0, 8.2 Hz), 7.02 (2H, ddd, *J* = 1.2, 7.0, 8.1 Hz), 6.90 (2H, dd, *J* = 1.3, 8.1 Hz), 6.83 (4H, ddd, *J* = 1.1, 6.8, 7.8 Hz), 6.74 (4H, dd, *J* = 1.7, 7.8 Hz), 6.43 (4H, ddd, *J* = 1.7, 6.8, 8.5 Hz), 6.32 (4H, dd, *J* = 1.1, 8.5 Hz), 6.08 (4H, s); ^13^C NMR (CDCl_3_): *δ*/ppm 147.13, 143.59, 137.58, 137.15, 136.80, 133.25, 131.63, 131.26, 127.29, 126.56, 125.63, 125.34, 121.29, 70.50; IR (KBr): *ν*/cm^−1^ 3049, 3030, 1952, 1591, 1491, 1457, 1441, 1426, 1306, 1285, 1172, 1159, 1071, 1053, 954, 880, 870, 797, 767, 746, 725; LR-MS (FD) *m*/*z* (%): 534.26 (11), 533.26 (45), 532.25 (M^+^, bp), 267.13 (2), 266.63 (10), 266.13 (22); HR-MS (FD) Calcd. for C_42_H_28_: 532.21910; Found: 532.22084; UV/Vis (CH_2_Cl_2_): λ_max_/nm (*ε*/M^−1^cm^−1^) 347 (9600), 290 (29,000), 246 (49,200).

#### 3.2.2. 5,5′-([1,1′-Biphenyl]-2,2′-diyl)bis(5*H*-dibenzo[*a*,*d*]cycloheptatrien-5-ylium) bis(tetrafluoroborate) **2**^2+^(BF_4_^−^)_2_

To a solution of diol **4** (252 mg, 445 µmol) in TFAA (4.7 mL) was added 42% HBF_4_ aq (670 µL, 4.45 mmol) at 0 °C to give a deep red solution, and the mixture was stirred for 2 h at 24 °C. The addition of dry ether led to precipitation of the dication salt. The precipitates were filtered and washed with dry ether three times to give **2**^2+^(BF_4_^−^)_2_ (267 mg) as a red powder with 85% yield.

Mp: 209 °C (decomp.); ^1^H NMR and ^13^C NMR spectra are identical to those of **2**^2+^(SbCl_6_^−^)_2_; IR (KBr): *ν*/cm^−1^ 3063, 1609, 1602, 1517, 1476, 1429, 1385, 1337, 1223, 1177, 1163, 1068, 897, 850, 836, 801, 781, 751, 735; LR-MS (FD) *m*/*z* (%): 548.22 (9), 534.23 (10), 533.23 (47), 532.22 (M^+^, bp), 531.22 (7), 266.61 (10), 266.11 (21); HR-MS (FD) Calcd. for C_42_H_28_: 532.21910; Found: 532.21844; UV/Vis (CH_3_CN): λ_max_/nm (∑/M^−1^cm^−1^) 567 (4550), 534 (4330), 432 (4360), 386 (6760), 313 (76900), 269 (26200).

#### 3.2.3. 5,5′-(1,1′-Biphenyl-2,2′-diyl)bis(5*H*-dibenzo[*a*,*d*]cycloheptatrien-5-ylium) bis(hexachloroantimonate) **2**^2+^(SbCl_6_^−^)_2_

To a solution of **1** (225 mg, 422 µmol) in dry CH_2_Cl_2_ (10 mL) was added (4-BrC_6_H_4_)_3_N^+●^SbCl_6_^−^ (680 mg, 833 µmol) at 23 °C, and the mixture was stirred for 1 h. The addition of dry ether led to precipitation of the dication salt. The precipitates were filtered and washed with dry ether three times to give **2**^2+^(SbCl_6_^−^)_2_·2(CH_2_Cl_2_) (500 mg) as a red powder in 86% yield. 

Mp: 258 °C (decomp.); ^1^H NMR (CH_3_CN): *δ*/ppm 8.98 (4H, brs), 8.49 (4H, brt, *J* = 7.0 Hz), 8.42 (4H, brt, *J* = 7.0 Hz), 7.80 (4H, brs), 7.61–7.53 (4H, m), 7.50 (4H, brs), 7.46 (2H, brt, *J* = 7.4 Hz), 7.06 (2H, brd, *J* = 7.4 Hz); ^13^C NMR (CH_3_CN): *δ*/ppm 180.00, 146.66, 145.95, 141.64, 140.49, 140.15, 139.40, 138.51, 136.97, 134.76, 134.14, 132.92, 130.99, 128.48; IR (KBr): *ν*/cm^−1^ 3055, 2931, 1636, 1600, 1512, 1473, 1426, 1383, 1335, 1253, 1222, 1164, 829, 847, 829, 796, 750, 730; LR-MS (FD) *m*/*z* (%): 534.26 (11), 533.26 (45), 532.25 (M^+^, bp), 267.13 (2), 266.63 (10), 266.13 (22); HR-MS (FD) Calcd. for C_42_H_28_: 266.10955 (M^2+^); Found: 266.10863 (M^2+^); UV/Vis (CH_3_CN): λ_max_/nm (∑/M^−1^cm^−1^) 569 (5000), 535 (4780), 425 (61,700), 387 (7740), 314 (85,600), 270 (43,000).

#### 3.2.4. Dispiro(dibenzo[*a*,*d*]cycloheptadiene-5,9′-phenanthrene-10′,5″-dibenzo[*a*,*d*]cycloheptadiene) **3**

To a solution of diol **5** in HFIP (3.0 mL) was added at 22 °C a solution of TMSClO_4_ in toluene (0.79 M, 1.40 mL, 1.11 mmol), which was prepared from TMSCl (500 µL, 3.96 mmol) and AgClO_4_ (830 mg, 4.00 mmol) in dry toluene (5 mL). After stirring at 22 °C for 1 h, the solvent was evaporated. To the residue were added dry CH_3_CN (8 mL) and activated zinc powder (1.78 g, 27.2 mmol). The mixture was stirred at 22 °C for 1 h, and then diluted with water. The whole mixture was extracted with CH_2_Cl_2_ three times. The combined organic layers were washed with water and brine, and dried over anhydrous Na_2_SO_4_. After filtration, the solvent was concentrated under reduced pressure. The crude product was purified by column chromatography on silica gel (hexane/CH_2_Cl_2_ = 5) to give **3** (47.5 mg) as a white solid with 33% yield.

Mp: 197–200 °C (decomp.); ^1^H NMR (CDCl_3_): *δ*/ppm 7.96 (2H, dd, *J* = 1.4, 8.2 Hz), 7.28 (2H, ddd, *J* = 1.2, 7.0, 8.2 Hz), 6.95–6.87 (6H, m), 6.78 (4H, d, *J* = 7.0 Hz), 6.44 (4H, brs), 6.29 (2H, dd, *J* = 1.2, 8.1 Hz), 6.11 (4H, brs), 2.92 (4H, dd, *J* = 9.7, 14.4 Hz), 2.57 (4H, brs); ^13^C NMR (CDCl_3_): *δ*/ppm 147.57, 145.21, 143.04, 137.89, 135.52, 1340.28, 129.31, 127.46, 126.55, 126.35, 123.55, 121.62, 71.24, 35.78; IR (KBr): *ν*/cm^−1^ 3106, 3053, 3019, 2936, 2843, 2360, 2341, 1920, 1673, 1657, 1597, 1486, 1441, 1430, 1385, 1365, 1314, 1280, 1265, 1217, 1162, 1129, 1100, 1083, 1068, 956, 866, 790, 781, 759, 746, 708, 668, 646, 625, 621, 589, 561, 534; LR-MS (FD) *m*/*z* (%): 539.26 (2), 538.26 (11), 537.26 (46), 536.26 (M^+^, bp), 268.13 (2); HR-MS (FD) Calcd. for C_42_H_32_: 536.25040, Found : 536.25269.

#### 3.2.5. 2,2′-Bis(5-hydroxydibenzo[a,d]cycloheptatriene-5-yl)biphenyl **4** [37]

To a solution of 2,2′-diiodobiphenyl [44] (1.13 g, 2.78 mmol) in dry THF (10 mL) was added *n*BuLi (1.55 M in hexane, 4.3 mL, 6.67 mmol) dropwise over 2 min at –78 °C. After stirring at –78 °C for 1 h, dibenzotropone (1.38 g, 6.67 mmol) was added to the suspension and the mixture was warmed to 23 °C. The resulting solution was stirred for 1 h at 23 °C, and then diluted with water. The whole mixture was extracted with EtOAc three times. The combined organic layers were washed with water and brine, and dried over anhydrous Na_2_SO_4_. After filtration, the solvent was concentrated under reduced pressure. The crude product was washed with methanol to give diol **4** (1.12 mg) as a white solid with 71% yield.

^1^H NMR (CDCl_3_): *δ*/ppm 8.13 (2H, dd, *J* = 1.2, 7.9 Hz), 7.50 (2H, ddd, *J* = 1.8, 6.8, 7.9 Hz), 7.39 (2H, dd, *J* = 1.3, 8.0 Hz), 7.30 (2H, dt, *J* = 1.2, 6.8 Hz), 7.02 (2H, dd, *J* = 1.8, 6.8 Hz), 6.98 (2H, dd, *J* = 1.3, 7.3 Hz), 6.86 (2H, dt, *J* = 1.3, 7.3 Hz), 6.79 (2H, ddd, *J* = 1.3, 7.3, 8.0 Hz), 6.67(4H, s), 6.61 (2H, ddd, *J* = 1.5, 7.5, 8.7 Hz), 6.46 (2H, dt, *J* = 1.2, 7.5 Hz), 6.39 (2H, dd, *J* = 1.2, 8.7 Hz), 6.05 (2H, dd, *J* = 1.5, 7.5 Hz), 5.04 (2H, s).

#### 3.2.6. 2,2′-Bis(5-hydroxydibenzo[a,d]cycloheptadiene-5-yl)biphenyl **5** [37]

To a solution of 2,2′-diiodobiphenyl (409 mg, 1.01 mmol) in dry THF (5 mL) was added *n*BuLi (1.55 M in hexane, 1.55 mL, 2.40 mmol) dropwise over 2 min at –78 °C. After stirring at –78 °C for 1 h, dibenzosuberone (432 µL, 2.40 mmol) was added to the suspension, and the mixture was warmed to 25 °C. The resulting solution was stirred for 15 h at 25 °C, and then diluted with water. The whole mixture was extracted with EtOAc three times. The combined organic layers were washed with water and brine, and dried over anhydrous Na_2_SO_4_. After filtration, the solvent was concentrated under reduced pressure. The crude product was washed with methanol to give **5** (467 mg) as a white solid with 81% yield.

^1^H NMR (CDCl_3_): *δ*/ppm 8.13 (2H, dd, *J* = 1.5, 7.6 Hz), 7.25 (2H, dt, *J* = 1.5, 7.6 Hz), 7.20 (2H, dt, *J* = 1.5, 7.6 Hz), 7.05 (2H, dd, *J* = 1.5, 7.6 Hz), 7.02 (2H, dd, *J* = 1.5, 7.6 Hz), 6.98–6.84 (8H, m), 6.66 (2H, dt, *J* = 1.5, 7.6 Hz), 6.44 (2H, dt, *J* = 1.4, 7.5 Hz), 6.00 (2H, dd, *J* = 1.4, 7.5 Hz), 5.15 (2H, s), 3.07–2.86 (4H, m), 2.70–2.60 (2H, m), 2.48–2.39 (2H, m).

#### 3.2.7. 5-(Spiro[dibenzo[*a*,*d*]cycloheptatriene-5,9′-fluoren]-4’-yl)-5*H*-dibenzo[*a*,*d*]cycloheptatriene-5-ylium hexachloroantimonate **8**^+^(SbCl_6_^−^)

A solution of **2**^2+^(SbCl_6_^−^)_2_ (83.9 mg, 69.8 µmol) in dry CH_3_CN (3 mL) was stirred for 2 days at 22 °C. The addition of dry ether led to precipitation of the monocation salt. The precipitates were filtered and washed with dry ether three times to give **8**^+^(SbCl_6_^−^) (56.7 mg) as a red powder with 94% yield.

Mp: 218 °C (decomp.); ^1^H NMR (acetone-*d*_6_): *δ*/ppm 9.71 (2H, s), 9.14 (2H, dd, *J* = 1.2, 8.1 Hz), 8.80 (2H, ddd, *J* = 1.0, 7.2, 8.1 Hz), 8.65 (2H, d, *J* = 8.9 Hz), 8.45 (1H, dd, *J* = 0.9, 7.7 Hz), 8.29 (2H, ddd, *J* = 1.2, 7.2, 8.9 Hz), 7.95 (1H, d, *J* = 7.7 Hz), 7.75 (1H, t, *J* = 7.7 Hz), 7.58 (2H, dd, *J* = 1.6, 7.3 Hz), 7.57 (1H, dd, *J* = 0.9, 7.7 Hz), 7.38 (2H, dt, *J* = 1.0, 7.3 Hz), 7.16 (2H, ddd, *J* = 1.6, 7.3, 8.3 Hz), 7.15 (2H, s), 7.13 (1H, dt, *J* = 0.9, 7.7 Hz), 7.03 (2H, dd, *J* = 1.0, 8.3 Hz), 6.68 (1H, dt, *J* = 0.9, 7.7 Hz), 5.78 (1H, d, *J* = 7.7 Hz); ^13^C NMR (acetone-*d*_6_): *δ*/ppm 181.17, 154.51, 154.39, 147.39, 145.88, 141.66, 141.23, 138.85, 138.12, 136.78, 136.75, 136.66, 136.02, 135.89, 134.09, 133.20, 132.88, 129.89, 128.78, 128.73, 128.46, 128.34, 128.16, 127.96, 127.84, 127.32, 122.72, 66.00; IR (KBr): *ν*/cm^−1^ 3061, 2935, 2859, 2360, 1600, 1513, 1479, 1428, 1385, 1337, 1258, 1177, 892, 848, 833, 801, 752, 734; LR-MS (FD) *m*/*z* (%): 588.24 (6), 566.19 (6), 533.23 (11), 532.23 (47), 531.22 (M^+^, bp); HR-MS (FD) Calcd. for C_42_H_27_: 531.21128; Found: 531.21124; UV/Vis (CH_2_Cl_2_): λ_max_/nm (∑/M^−1^cm^−1^) 567 (5200), 533 (4680), 406 (6310), 388 (7980), 315 (104,500), 275 (35,800), 242 (46,700).

### 3.3. Crystal Data

Data were collected with a Rigaku Mercury 70 diffractometer (Mo-Kα radiation, *λ* = 0.71075 Å). The structure was solved by the direct method (SIR2004) and refined by the full-matrix least-squares method on *F*^2^ with anisotropic temperature factors for non-hydrogen atoms. All the hydrogen atoms were located at the calculated positions and refined with riding.

**1**: Crystals were obtained by recrystallization from CHCl_3_/hexane. MF: C_42_H_28_, FW: 532.68, colorless block, 0.20 × 0.20 × 0.20 mm^3^, monoclinic *P*2_1_/*c*, *a* = 13.3661(12) Å, *b* = 12.6558(11) Å, *c* = 16.166(2) Å, *β* = 95.8567(13)°, *V* = 2720.4(5) Å^3^, *ρ* (Z = 4) = 1.301 g cm^−3^. A total of 20359 reflections (2*θ*_max_ = 55.0°) were measured at *T* = 150 K. Numerical absorption correction was applied (*µ* = 0.735 cm^−1^). The final *R1* and *wR2* values are 0.0439 (I > 2*σ*I) and 0.1179 (all data) for 5332 reflections and 379 parameters. Estimated standard deviations are 0.0017–0.003 Å for bond lengths and 0.09–0.17° for bond angles. CCDC 1580196.

**2**^2+^(SbCl_6_^−^)_2_·2(CH_2_Cl_2_): Crystals were obtained by recrystallization from CH_2_Cl_2_/ether. MF: C_44_H_32_Cl_16_Sb_2_, FW: 1371.48, red platelet, 0.60 × 0.10 × 0.10 mm^3^, monoclinic *P*2_1_/*c*, *a* = 11.109(2) Å, *b* = 15.074(3) Å, *c* = 30.901(6) Å, *β* = 91.693(3)°, *V* = 5172(2) Å^3^, *ρ* (Z = 4) = 1.761 g cm^−3^. A total of 31500 reflections (2*θ*_max_ = 55.0°) were measured at *T* = 150 K. Numerical absorption correction was applied (*µ* = 19.007 cm^−1^). The final *R1* and *wR2* values are 0.0983 (I > 2*σ*I) and 0.2428 (all data) for 8942 reflections and 559 parameters. Estimated standard deviations are 0.004–0.03 Å for bond lengths and 0.13–0.8° for bond angles. CCDC 1580197.

**3**: Crystals were obtained by recrystallization from benzene/hexane. MF: C_42_H_32_, FW: 536.71, colorless prism, 0.40 × 0.30 × 0.20 mm^3^, monoclinic *P*2_1_/*n*, *a* = 16.557(6) Å, *b* = 20.113(7) Å, *c* = 17.343(6) Å, *β* = 103.513(5)°, *V* = 5616(4) Å^3^, *ρ* (Z = 8) = 1.270 g cm^−3^. A total of 42292 reflections (2*θ*_max_ = 55.0°) were measured at *T* = 150 K. Numerical absorption correction was applied (*µ* = 0.716 cm^−1^). The final *R1* and *wR2* values are 0.0679 (I > 2*σ*I) and 0.1926 (all data) for 11017 reflections and 757 parameters. Estimated standard deviations are 0.003–0.005 Å for bond lengths and 0.15–0.3° for bond angles. CCDC 1580198.

**8**^+^(SbCl_6_^−^)·(CH_3_CN): Crystals were obtained by recrystallization from CH_3_CN/ether. MF: C_44_H_30_Cl_6_NSb, FW: 907.20, red platelet, 0.80 × 0.20 × 0.10 mm^3^, triclinic *P*$\bar{1}$, *a* = 9.518(7) Å, *b* = 13.5559(10) Å, *c* = 15.433(11) Å, *α* = 98.195(9)°, *β* = 92.202(13)°, *β* = 102.889(11)°, *V* = 1916(2) Å^3^, *ρ* (Z = 2) = 1.572 g cm^−3^. A total of 14621 reflections (2*θ*_max_ = 55.0°) were measured at *T* = 150 K. Numerical absorption correction was applied (*µ* = 11.707 cm^−1^). The final *R1* and *wR2* values are 0.0415 (I > 2*σ*I) and 0.1049 (all data) for 7454 reflections and 454 parameters. Estimated standard deviations are 0.0013–0.007 Å for bond lengths and 0.04–1.2° for bond angles. CCDC 1580199.

## 4. Conclusions

Based on a molecular design that maximizes the effects of TBI, the novel Ar_4_DHP **1** with two spiro(dibenzocycloheptatriene) units was designed as an electron-donating hydrocarbon. Based on reversible interconversion with the bis(dibenzotropylium)-type dicationic dye **2**^2+^ accompanied by C–C bond formation/cleavage (*dyrex* behavior), the present redox pair exhibits electrochromism with a vivid change in color, and thus represents a class of less well-developed hydrocarbon-based systems. Compound **1** was predicted to have a highly strained structure during the molecular design by a DFT calculation, which was finally confirmed by X-ray analysis of the hydrocarbon prepared as a stable entity. A more highly strained structure was observed in the tetrahydro derivative **3**, the C_9_–C_10_ bond length of which is the greatest among the values ever reported for Ar_4_DHPs. Thus, the spiro(dibenzocycloheptadiene) unit was proven to be a promising spiro skeleton for inducing extreme bond expansion when incorporated in the clamped/fused HPE skeleton.

## Supplementary Materials

Supplementary materials are available online, Figure S1: Change in the UV/Vis spectrum upon electrochemical reduction (20 *µ*A) of as-prepared **2**^2+^ in CH_2_Cl_2_ containing 0.05 M Bu_4_NBF_4_ as a supporting electrolyte (every 4 min).
