# Supplementary material for: 9,10-Dihydrophenanthrene with Two Spiro(dibenzocycloheptatriene) Units: A Highly Strained Caged Hydrocarbon Exhibiting Reversible Electrochromic Behavior
Source: Molecules. 2017 Nov 4;22(11):1900. doi: 10.3390/molecules22111900 (PMC6150351; doi:10.3390/molecules22111900)
Supplement: Supplementary file 1 [file molecules-22-01900-s001.pdf]

Supplementary

9,10-Dihydrophenanthrene with Two Spiro(dibenzocycloheptatriene) Units: A Highly Strained Caged Hydrocarbon Exhibiting Reversible Electrochromic Behavior

Yusuke Ishigaki*, Yuki Hayashi, Kazuma Sugawara, Takuya Shimajiri, Wataru Nojo, Ryo Katoono and Takanori Suzuki*

Department of Chemistry, Faculty of Science, Hokkaido University, Sapporo 060-0810, Japan; gaudryceras@eis.hokudai.ac.jp (Y.H.); kazuama_ns0210@eis.hokudai.ac.jp (K.S.); t.shimajiri@sci.hokudai.ac.jp (T.Sh.); nojo@sci.hokudai.ac.jp (W.N.); katoono@sci.hokudai.ac.jp (R.K.)

*Correspondence: yishigaki@sci.hokudai.ac.jp (Y.I.); tak@sci.hokudai.ac.jp (T.Su.);
Tel.: +81-11-706-2701 (Y.I.); Tel.: +81-11-706-2714 (T.Su.)

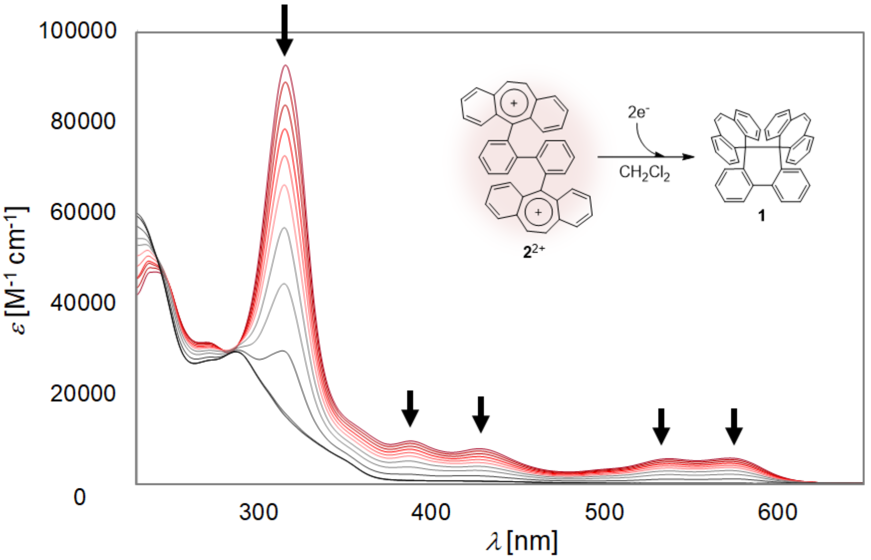

Figure S1. Change in the UV/Vis spectrum upon electrochemical reduction (20 μA) of as-prepared **2**$^{2+}$ in CH_2Cl_2 containing 0.05 M Bu_4NBF_4 as a supporting electrolyte (every 4 min).
